# Feasibility, Acceptability, and Preliminary Impacts of Web-Based Patient Education on Patients With Schizophrenia Spectrum Disorder: Quasi-Experimental Cluster Study

**DOI:** 10.2196/13073

**Published:** 2019-10-17

**Authors:** Anna Laine, Maritta Välimäki, Virve Pekurinen, Eliisa Löyttyniemi, Mauri Marttunen, Minna Anttila

**Affiliations:** 1 Department of Nursing Science University of Turku Turku Finland; 2 School of Nursing Hong Kong Polytechnic University Hong Kong Hong Kong; 3 Department of Biostatistics University of Turku Turku Finland; 4 Institute of Criminology and Legal Policy` University of HelsinkiHelsinkiFinland; 5 Helsinki University Hospital Helsinki Finland

**Keywords:** mental health, patient education, schizophrenia, feasibility study, internet, information system

## Abstract

**Background:**

Web-based interventions are promising tools for increasing the understanding of illness and treatment among patients with serious mental disorders.

**Objective:**

This study aimed to test the feasibility and acceptability of a Web-based patient education intervention using a quasi-experimental cluster design to report feedback on patient education sessions and the website used and to report preliminary evidence of the intervention’s impact on patients with schizophrenia spectrum disorder.

**Methods:**

A single-blind, parallel, quasi-experimental cluster study over a 6-month period comparing Web-based education (n=33) with a nonequivalent control group (treatment as usual, n=24) for people with schizophrenia spectrum disorder was conducted. Participants (N=57) were recruited from one psychiatric hospital (6 wards). Feasibility was assessed by participants’ commitment (refusal rate, dropout rate) to the study. Acceptability was assessed as participants’ commitment to the intervention. Patient education sessions and website feedback were assessed by the patients and health care professionals. The preliminary impact of the sessions on patients’ self-efficacy, self-esteem, illness cognition, and knowledge level was measured at baseline and follow-ups (8 weeks, 6 months) with self-rated questionnaires.

**Results:**

The refusal rate among patients was high with no statistically significant difference (69% [74/107] in the intervention group, 76% [76/100] in the control group; *P*=.21). The same result was found for the dropout rates (48% [16/33] vs 58% [14/24]; *P*=.46). The acceptability of the intervention was good; 31 participants out of 33 (94%) completed all five sessions. Feedback on the intervention was mainly positive; three out of four subscales of session were rated above the midpoint of 4.0. Feedback on the website was also positive, with a grade of good for content (69%, 20/29 patients; 75%, 21/28 professionals), layout (62%, 18/29 patients; 61%, 17/28 professionals), and usability (62%, 18/29 patients; and 68%, 19/28 professionals). The patients using the intervention had significantly higher scores 6 months after the sessions in self-efficacy (baseline mean 26.12, SD 5.64 vs 6-month mean 29.24, SD 6.05; *P*=.003) and regarding knowledge level about schizophrenia (mean 11.39, SD 4.65 vs 6-month mean 15.06, SD 5.26; *P*=.002), and lower scores in the subscale of helplessness in illness cognition (mean 2.26, SD 0.96 vs 6-month mean 1.85, SD 0.59; *P*=.03). Differences from the control group were not significant. No differences were found in patients’ self-esteem or other subscales in illness cognition.

**Conclusions:**

The patients were reluctant to participate in the study and tended to drop out before the follow-ups. Once they had participated, their acceptance of the intervention was high. A more effective recruitment strategy and monitoring method will be needed in future studies. To assess the impact of the intervention, a more rigorous study design with an adequately powered sample size will be used in cooperation with outpatient mental health services.

## Introduction

### Background

Schizophrenia, with an age-standardized point prevalence of 0.28% [1], is one of the most severe mental disorders that causes comprehensive impairments to a person’s cognitive functioning, requiring long-term treatment [2]. For patients with schizophrenia, nonadherence in treatment is a remarkable problem [3-5] owing to feelings of being stigmatized or self-stigmatized [6]. Continuous symptoms and lack of insight [2] often cause patients to relapse and increase their hospitalizations [7]. Therefore, a variety of psychosocial interventions have been developed for patients with schizophrenia to increase treatment adherence and improve quality of life [2]. Dropout rates in psychosocial interventions have been found to be low [8].

Psychosocial interventions, such as patient education, seem to reduce relapses, readmission, medical nonadherence, and the length of hospital stays of patients with schizophrenia [9]. Various sets of guidelines have also recommended patient education for persons with schizophrenia [2,10]. Patient education includes guidance and information about the illness and how to cope with it. The education can be realized individually or in small groups of patients and their relatives with different kinds of supportive material, such as written procedures, videos, and Web-based programs [9,11]. Previous studies have shown that patients with mental health problems search out health care knowledge on the internet [12-16], which justifies using Web-based interventions in mental health care [12,17].

A variety of Web-based patient education interventions for patients with mental health problems exist, for example, for patients with depression [18-21], bipolar disorder [22], and schizophrenia [23-25]. Studies have shown that Web-based patient education has improved patients’ compliance with medication [11] and reduced symptoms [23,26]. Web-based patient education has been found to improve patients’ knowledge levels about their illness [23,27]. However, on the basis of Cochrane review by Välimäki et al [11], there is no difference in improvement when compared with other psychoeducational methods used for patients with schizophrenia spectrum disorder.

### Objectives

On the basis of the Cochrane review [11], Web-based patient education for patients with schizophrenia is as effective as patient education carried out traditionally, for example, with leaflets, when comparing patients’ satisfaction in treatment, even though the number of studies asserting this claim is small and many of them are underpowered. Previous studies have also found that information related to serious mental disorders described on the internet is often low in quality [28]. This finding is concerning because many people with serious mental disorders use the internet as a source of information [16,17]. Therefore, it is critical that online mental health literacy [16] and Web-based interventions [19] are developed for these specific groups, even though it seems that people with mental disorders use the internet less frequently [16,17] compared with the general population [29]. Previous studies have revealed a number of obstacles, such as high refusal and dropout rates [30], although other results have found high acceptability rates [31] and engagement regarding Web- and mobile-based interventions [23,32-35]. On the other hand, Killikelly et al [36] found, in their systematic review, that participants’ adherence to Web- and mobile-based interventions ranged between 28% and 100%. Indeed, persons with schizophrenia can be a challenging target group for study recruitment in general [30,37-40]. The aim of this study was to test the feasibility and acceptability of a Web-based educational intervention among persons with schizophrenia using a quasi-experimental study design and to report preliminary evidence of its impact on self-efficacy, self-esteem, knowledge level about schizophrenia, and illness cognition.

## Methods

### Design and Sample

A single-blind, parallel, quasi-experimental cluster study design was used with a nonequivalent control group for an 8-week timeframe and a 6-month follow-up to evaluate possible short-term and long-term impacts [41] of the intervention. Long-term impacts were included in the assessment as is recommended by the World Health Organization when testing novel digital health interventions [42]. A cluster study design is usable when aiming to avoid information and experience flow between individual participants in intervention and control groups [43,44], as information and experience flow between study groups could have effects on the study results [45,46].

### Setting, Eligibility Criteria, and Recruitment

The study was run between May 2015 and May 2016 in 1 psychiatric hospital (6 wards) in Southern Finland. A total of 3 wards caring particularly for patients with schizophrenia spectrum disorder who showed interest in participating in the feasibility study were purposefully invited (a total of 41 beds, 2 closed rehabilitation wards, and 1 closed acute ward treating patients with schizophrenia) and assigned to be the intervention wards. A total of 3 other corresponding wards were then purposefully selected and invited to join the study owing to their match with the intervention wards (a total of 44 beds, 2 closed rehabilitation wards, and 1 closed acute ward treating patients with schizophrenia) when randomization of participants was not reasonable [46], given the possible information flow between the participants [44].

Patients were eligible to participate if they had been admitted to the study ward during the data collection period, were 18 years old or older, had a primary diagnosis of schizophrenia spectrum disorder (F20–F29, International Classification of Diseases, 10^th^ Revision [ICD-10]) [47], were able to write, read, and speak Finnish, and had volunteered to participate in the study with a written informed consent. Exclusion criteria were an unclear diagnosis, a short hospital period (less than 1 week, which does not allow a proper informed consent process or time to run the intervention), an impaired mental state (assessed by the staff members based on their daily experiences), or a lack of willingness to participate in the study. In addition, if the patient was discharged and rehospitalized during the study period, he or she could only participate in the study once.

One staff member in each study ward with access to hospital medical records acted as a contact person. The contact person was responsible for the patient recruitment and assessment of whether the patients fulfilled the inclusion criteria. The contact person monitored that the rehospitalized patients were not recruited again. The contact person informed the eligible patients about the study (orally and in written format) and further informed the researcher (AL) about the potential participants. Potential participants were informed that they had the possibility to meet the researcher if they wanted more detailed information about the study (voluntary participation, confidentiality, withdrawal without any penalty, or consequences to care) in addition to the contact person’s information and other written information. If the patients were willing to participate in the study, they signed 2 informed consent forms [48,49] and provided the baseline data. The completed forms were then sealed in an envelope.

The researcher visited the study wards weekly to ensure that the protocol for patient recruitment was followed and eligible patients were invited to participate in the study. For the follow-up, the instruments were distributed to the patients during their hospital stay (long-term patients) or were sent by post to discharged patients (only with patient permission). The sample size was determined by how many participants were willing and eligible to participate during the 6-month period.

The study was assessed by the Ethics Committee of the Hospital District of Southwest Finland (ETMK:40/1801/2015). The research permission committee of the study organization granted permission for data collection on the study wards. The outcome assessment was carried out according to ethical guidelines [48-50] and the Finnish legislation concerning research [51] and personal data registration [52].

### Study Groups

#### Intervention Group

Patients in the intervention group received Web-based educational intervention by using a health-related website, MentalNet. This website has been originally designed for adult patients with psychosis (ICD-10 codes F20-F29 [47]) to increase their understanding of their illness and its treatment [53]. In its current version, the website targets patients, their relatives, and health care professionals. The website is secured and accessible only with passwords. The website includes the following components: educational material, a discussion forum, and a question and answer column [53,54]. The main component is the educational reading material [23], which includes information for patients with schizophrenia spectrum disorder divided into 5 themes, and tens of accurate and high-quality website links related to each of the 5 topics. There are also tasks for the patients and audio-recorded success stories to increase patients’ knowledge about their disorder [9]. The tasks are related to information themes, which were formed according to patients’ interests during the patient education sessions. The content of the educational material is described in Table 1. The website has a Health On the Net Code of Conduct certificate as a trustworthy and reliable medical website [55].

The intervention was carried out by health care professionals (nurses, psychologists, and occupational therapists) working in the intervention wards and who were trained to run the patient education sessions and to use the website with the patients [56]. A detailed description of the professionals and Web-based training for professionals to run the patient education sessions are reported elsewhere [56]. The professionals had permanent working positions only in their own intervention or control ward. The intervention included 5 sessions with specific topics based on the information themes of the educational materials on the website: (1) mental disorder, (2) treatment, (3) well-being, (4) patients’ rights, and (5) daily life. The professionals scheduled sessions with the patients once per week (each session about 45–60 min); with the exception that if the inpatient stay was planned to be shorter than a week, then a tighter schedule was made (eg, once a day). The professionals also prepared the material needed for each session (computer, internet connection, printer, and a peaceful place). To ensure patient orientation, the order of the themes was not set in advance. Instead, in each session, 1 topic was selected based on the patient’s preference. The patient was encouraged to identify any questions or concerns they may have related to the selected topic to discuss with the professional and to use the website to find the answers to his or her questions. The role of the professional was to help the patient focus on questions important to him or her, to help the patient use the website, and to search for information and answers to his or her questions.

**Table 1:** Content of educational material of MentalNet.

Theme	Information topics	Tasks
Mental disorder	Impact of the disorder on person with the disorder Impact of the disorder on caregivers Different types of psychosis (F20–F29, International Classification of Diseases, 10^th^ Revision) Depression	Measures related to mental health
Treatment	Care and rehabilitation Life point when treatment is needed Places where the treatment is realized Health care professional participating in the treatment Practical aspects related to treatment Examinations Different types of treatment Restrictive practice	Time management diary
Well-being	Mental health Nutrition Physical training Sleep Hygiene Intoxicants and smoking Family and relationships Sexuality Work Education Spare time Spiritual well-being	Healthy eating plate Physical activity pie Sleeping diary Tests about using intoxicants Social network circle Relaxing exercise
Patient’s rights	Adequate treatment Fair treatment Self-determination The right to information Review of documents Data protection Involuntary treatment Patient ombudsman Patient injury Laws related to patient’s rights	Questions about patient’s rights
Daily life	Economic support A guardian Living Support for taking care of home Return to work Support from fellow man	Test related to instrumental activities of daily living

#### Control Group

Patients in the control group continued their treatment with care as usual. They did not receive any psychoeducational intervention provided by the researcher or have access to the website.

### Outcomes and Assessment Instruments

#### Primary Outcomes

##### Feasibility

Feasibility was assessed by patient refusal of the study (yes, no), participation in the follow-ups (yes, no), and whether they dropped out of the study (attrition rate).

##### Acceptability

Acceptability was assessed by the patients in the intervention group. The number of patients participating in all possible sessions (5 sessions per participant) and the total amount of sessions were calculated.

##### Feedback of the Patient Education Sessions

The one-on-one patient education sessions were assessed by both the patient and the professional using the Finnish translation of the Session Evaluation Questionnaire (Form 5) (SEQ) [57]. SEQ is a self-rating instrument originally designed to measure psychotherapy and counselling sessions and to measure participants’ (client and/or therapist) moods after the session. The SEQ includes 21 bipolar adjective items divided into 2 parts: 11 items about the session itself (1 global item *bad–good*, 5 subscale items for *depth*, and 5 subscale items for *smoothness*) and 10 items about participants’ moods after the session (5 subscale items for *positivity* and 5 subscale items for *arousal*), such as “This session was *valuable-worthless*, *easy-difficult*” or “Right now I feel *happy-sad*, *angry-pleased.*” The range of the scale is 1 to 7, with a midpoint of 4.00. The mean scores of each subscale items are calculated to form a subscale score, and mean values above the midpoint of 4.00 are considered to be a positive evaluation of the session [58]. In previous studies, the internal consistency of the instrument has been found to be relatively good (Cronbach alpha .63-.93) [58]. In our study, Cronbach alphas ranged between .34 and .84 (patients: depth .67, smoothness .73, positivity .73, arousal .39; and professionals: depth .81, smoothness .75 positivity .84, arousal .34). The instrument was translated into Finnish with a back-translation method [59,60] using an independent professional translator and the original developer of the questionnaire.

##### Feedback of the Website

Feedback about the MentalNet website was collected from the patients and the professionals, after all 5 sessions were completed, using a 5-point Likert scale (very good–very poor) and with the possibility to give written feedback. The feedback targeted the content, layout, and usability of the website to ensure that the website is usable and meets the needs of its users [61,62].

#### Secondary Outcomes at Baseline, 8 Weeks, and 6-Month Follow-Up

##### Self-Efficacy

The General Self-Efficacy Scale (GSE) [63] is a widely used self-rating instrument designed to measure the general sense of perceived self-efficacy in different types of difficult life events. The instrument contains 10 items, and its responses are in the form of a 4-point Likert scale. The sum score of the responses ranges from 10 to 40; a higher score represents greater sense of self-efficacy. In a study by Scholz et al [64], the psychometric properties of the GSE were examined in 25 countries, and the Cronbach alpha varied from .75 to .91. In our study, the Cronbach alpha value varied between .92 and .96.

##### Self-Esteem

The Rosenberg Self-Esteem Scale (SES) [65] is a self-rating instrument designed to measure overall self-esteem. It includes 10 items with a 4-point Likert scale. The sum score of the answers ranged from 10 to 40; higher scores indicate higher self-esteem. The SES has been translated into at least 28 languages and is widely used in many countries. In a review by Schmitt and Allik [66], the data of SES from 53 countries were compared. Internal consistency was found to be good (Cronbach alpha .80, range .45 to .90). In our study, the Cronbach alpha value varied between .83 and .90.

##### Illness Cognition

The Illness Cognition Questionnaire [67] is a self-rating instrument designed to measure illness cognition of people with chronic illnesses as how they perceive and think about their illness. The participants are asked to assess to what extent they assess with 18 statements of the instrument by using a 4-point Likert scale (1=not at all, 2=somewhat, 3=to a large extent, and 4=completely). The instrument consists of 3 subscales (6 items each) measuring a basic set of illness cognitions that includes both unfavorable (negative) and favorable (positive) ways of adjusting to chronic disease: *helplessness* (with negative perspective, eg, “My illness limits me in everything that is important to me.”), *acceptance* (with positive perspective, eg, “I can handle the problems related to my illness.”), and *perceived benefits* (with a positive perspective, eg, “I have learned a great deal from my illness.”). Internal consistency has proven to be adequate when using the instrument, for example, with patients with chronic pain (Cronbach alpha for helplessness .88, acceptance .91, and perceived benefits .83) and chronic fatigue (Cronbach alpha for helplessness .83, acceptance .90, and perceived benefits .81) [68]. In this study, the Cronbach alpha varied between .77 and .97 for helplessness, .74 and .91 for acceptance, and .61 and .91 for perceived benefits. The instrument was translated into Finnish with a back-translation method [59,60] using an independent professional translator and the original developer of the questionnaire.

##### Knowledge Level

Knowledge about Schizophrenia Questionnaire [69] is a self-rating instrument designed to measure the knowledge of patients with schizophrenia about their illness and its management. The instrument is a multiple-choice test with 25 items with themes as follows: diagnosis, frequency, etiology, progress and prognosis of illness, medication and its side effects, drug-free treatments, stress, and legislation. The respondent is given 1 point for each correct answer, and the sum score ranges between 0 and 25; a higher score represents a high knowledge level. Internal consistency of the instrument is proven to be good (Cronbach alpha .75 [69]). In this study, the Cronbach alpha varied between .71 and .81. The instrument was translated into Finnish with a back-translation method [59,60] using an independent professional translator and the original developer of the questionnaire. A cultural modification was made for question number 20 to fit the Finnish health care system and the Mental Health Act about involuntary treatment [70].

#### Sociodemographic Information and Internet Use at Baseline

Information about the patients’ age, gender, age at first contact with psychiatric services, attitudes toward computers or the internet, and their computer or internet skills was collected. Attitudes toward computers or the internet and their computer or internet skills were assessed with a 5-point Likert scale (*Your attitude toward using the computer/internet is [1=very positive to 5=very negative*] and *Your computer/internet skills are [1=very good to 5=poor*]). Moreover, the adapted instrument [16] by Choi and DiNitto [71] was used to describe participants’ internet use and purpose of internet use. The options *communicate with health professionals about health-related issues* and *communicate with other users about health-related issues* were added [16] to the original questions about internet use [71].

### Data Analysis

Descriptive statistics were used for numerical variables with a median, mean, and standard deviation (SD), whereas categorical variables are reported with counts and percentages, and sum scores for each scale were calculated. Feedback from the patients and the professionals regarding the MentalNet website were compared using a Chi-square test (*x^2^*). Data regarding self-efficacy, self-esteem, illness cognition, and knowledge level were analyzed with hierarchical linear mixed models, allowing subjects to have missing values. The analysis included all 3 time points (baseline, 8 weeks, and 6 months). The model was adjusted by age, gender, and group of participation. One main interest was to focus on whether the mean change between time points differed among the groups. A compound symmetry covariance structure was used for repeated measures. A Cronbach alpha was calculated for all questionnaires. These statistical tests were performed as 2-tailed, with a significance level set at .05. The analyses were performed using the SAS System, version 9.4 for Windows (SAS Institute Inc.).

Cohen *d* was calculated between baseline and 6 months to find out the effect size of the intervention on patients’ self-efficacy, self-esteem, illness cognition, and knowledge level. Based on Cohen, 0.2 is considered a small, 0.5 is a medium, and 0.8 is a large effect size [72]. The analysis was performed using an online calculator [73].

## Results

### Sociodemographic Information

In both the intervention and control groups, the mean age of the patients was approximately 41 years (range 20–66 in the intervention group and 23–69 in the control group). Their age when they first accessed mental health care services was around 24 years (range 8–47 in the intervention group and 3–68 in the control group). The proportion of women in the intervention group was twice as much as it was in the control group.

Although the patients’ internet skills varied, about 70% reported that they used the internet and about 40% had good internet skills (very good or good). A clear majority had positive attitudes toward computers. Patients used the internet most often for searching for knowledge other than health information, emailing, banking, reading news or books, and social media. Background information about patients and their use of the internet are presented in more detail in Table 2.

**Table 2:** Background information of the patients.

Patient’s information		Intervention group (N=33)	Control group (N=24)
Age (years), mean (SD)		42 (14.13)	41 (12.90)
Age (years) when first receiving mental health care, mean (SD)		24 (9.30)	24 (12.60)
**Gender, n (%)**			
	Female	18 (55)	6 (25)
	Male	15 (45)	18 (75)
**Use of internet, n (%)**			
	Never user	6 (18)	4 (17)
	Previous user	4 (12)	3 (13)
	Current user	23 (70)	17 (71)
**Computer/internet skills, n (%)**			
	Very good	3 (9)	4 (17)
	Good	10 (30)	6 (25)
	Neither good nor bad	6 (18)	7 (29)
	Fairly poor	1 (3)	2 (8)
	Poor	13 (39)	5 (21)
**Attitudes toward computers/internet, n (%)**			
	Very positive	8 (24)	8 (33)
	Positive	15 (45)	10 (42)
	Neither positive nor negative	7 (21)	4 (17)
	Negative	3 (9)	2 (8)
	Very negative	0 (0)	0 (0)
**Purpose of the internet use, n (%)**			
	Research information about other topics of interest	15 (45)	16 (67)
	Send/receive email	14 (42)	15 (63)
	Watch videos	11 (33)	15 (63)
	Do banking online and/or pay bills	11 (33)	14 (58)
	Read newspapers, magazines, and books online	7 (21)	16 (67)
	Use social networking or dating site	9 (27)	14 (58)
	Research health-related information	10 (30)	10 (42)
	Play games online	8 (24)	11 (46)
	Buy products online	5 (15)	11 (46)
	Communication with others	5 (15)	3 (13)
	Other	3 (9)	6 (24)
	Communication with health professionals	2 (6)	3 (13)

### Feasibility

**Figure 1:**
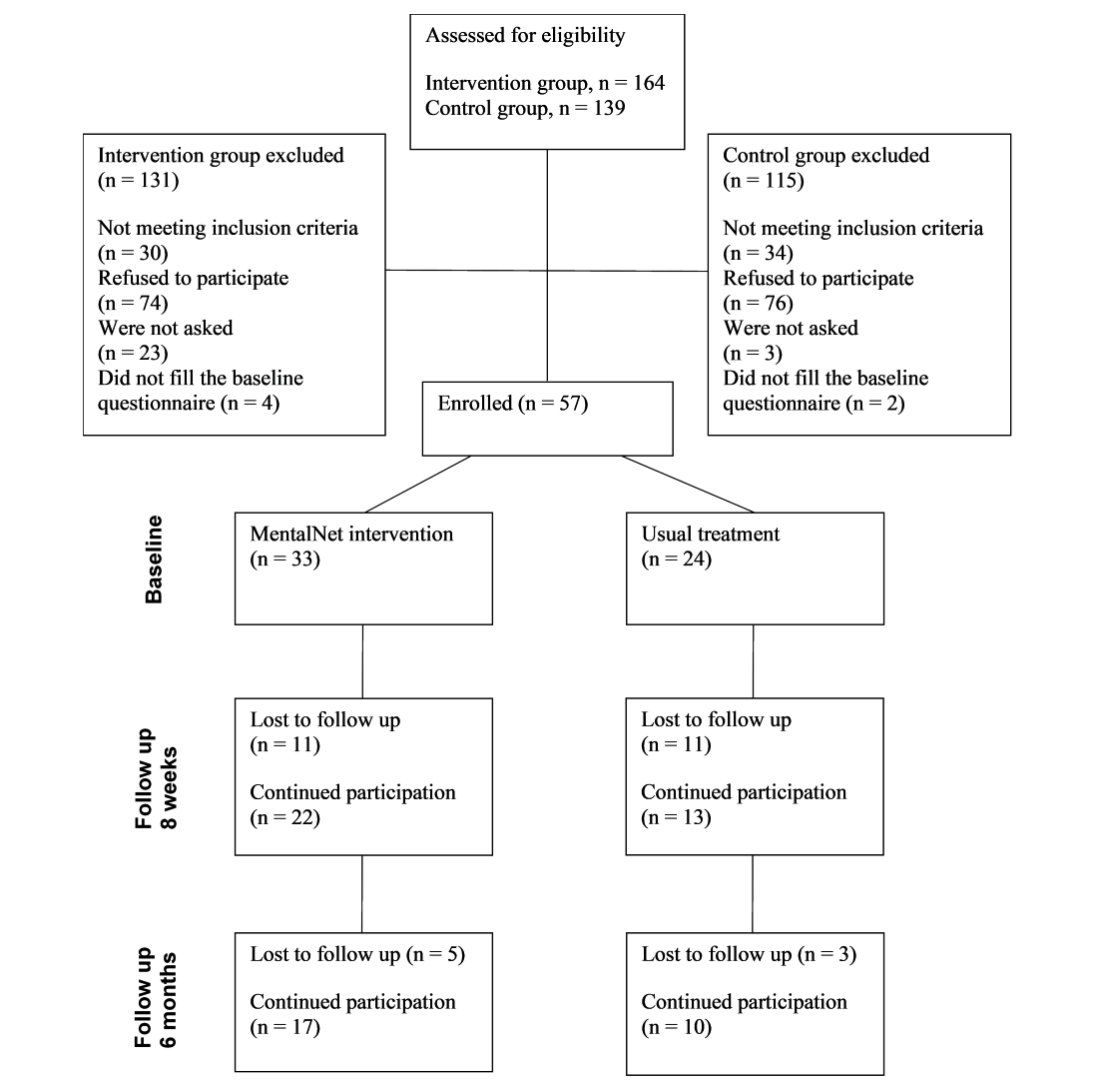
Flow diagram of patients.

A flow diagram of participating patients is presented in Figure 1. During the data collection, 303 patients were assessed for eligibility. A total of 213 patients were invited to participate in the study and 150 of them refused. The refusal rate between the intervention and control groups was not statistically significant (69% [74/107] in the intervention group vs 76% [76/100] in the control group, *P*=.21). At baseline, out of the allocated patients, 58% (33/57) were in the intervention group and 42% (24/57) in the control group. After baseline, 33% (11/33) dropped out of the intervention group and 46% (11/24) dropped out of the control group. In total, 8 patients dropped out after the first follow-up: 5 from the intervention group and 3 from the control group. This left us with a total of 63% (17/27) patients in the intervention group and 37% (10/27) in the control group. The difference in the number of dropouts between the intervention and control groups was not statistically significant (48% [16/33] vs 58% [14/24]; *P*=.46). No statistically significant differences between completers and dropouts were found regarding their gender, age, or age at first time of received mental health care (see Table 3).

### Acceptability of the Intervention

It was planned that each study participant (N=33) would have 5 intervention sessions. Out of 33 participants, 31 patients (94%) had all 5 sessions of the intervention. Out of the remaining 2 patients, 1 patient completed 3 sessions and 1 patient 1 session. Altogether, 159 (96%) sessions out of 165 planned sessions were realized.

### Feedback of the Patient Education Sessions

Each of the 5 patient education sessions were evaluated by the patients and the professionals directly afterward. The means of the global item *bad-good* and the subscales *depth*, *smoothness*, and *positivity* were evaluated to be above the midpoint of 4.0 (with a range of 1–7) by both the patients and professionals. Patients’ evaluations were more positive with a statistically significant difference in the global item *bad-good* (*P*=.02) and in the subscale *depth* (*P*=.04), and professionals’ evaluations were more positive with a statistically significant difference in the subscale *positivity* (*P*=.03) when evaluations were compared with each other (see Table 4).

**Table 3:** Demographic characteristics of patients who dropped out and completed the study.

Demographic characteristics	Dropouts (N=30)	Completers (N=27)	*P* value
Age (years), mean (SD)	40 (14.33)	43 (12.61)	.30
Age (years) when first received mental health care, mean (SD)	24 (10.08)	23 (11.49)	.27
Gender (female), n (%)	11 (37)	14 (52)	.38

**Table 4:** Session evaluation of Web-based patient education meetings by patients and health care professionals combined from all 5 sessions (n=number of sessions) based on dimensions of Session Evaluation Questionnaire [58].

Dimension	Patients (N=33)			HCPs^a^ (N=33)			*P* value
	n	Mean (SD)	Median	n	Mean (SD)	Median	
Bad-good	154	5.51 (1.49)	6.00	152	5.13 (1.40)	6.00	.02^b^
Depth	150	4.57 (1.15)	4.60	146	4.31 (0.96)	4.20	.04^b^
Smoothness	151	4.99 (1.12)	5.00	146	5.00 (0.85)	5.00	.98
Positivity	149	5.27 (1.17)	5.40	138	5.54 (0.82)	5.80	.03^b^
Arousal	153	3.47 (0.94)	3.60	138	3.33 (0.58)	3.40	.12

^a^HCPs: health care professionals.

^b^Statistically significant difference.

### Feedback of the Website

The patients and the professionals gave their feedback on the website. Most (≥65%) of the patients and the professionals responded that the content, layout, and usability of MentalNet was good or very good. The numerical feedback is presented in more detail in Table 5.

In the written feedback, patients expressed thanks for the opportunity to participate in the intervention, which they had found meaningful. Patients were satisfied with the comprehensive and good content of the website. They had been able to get important information that had helped them to understand their situation and would be valuable in the future. Critical feedback from patients was related to tasks that could be too difficult if the patient lacked the computer skills and if the links were not working.

The professionals offered, in their insight, that the content of the website included comprehensive information with good themes. In their opinion, using the website gave a structure for patient education. The professionals felt that these themes with new or iterated information for patients was important to go through, and the professionals expressed that they would use the website again in the future. The professionals were also able to gain new information about patients. This information could be used for better care of the patients in the future. In their opinion, the website was useful and easy to use with those patients who were enthusiastic about the intervention, able to use the website, and willing to find information independently. On the other hand, some professionals felt that going through the website was useless and it was hard to get patients interested in using it. The professionals gave critical feedback about the content and layout of the website. In their opinion, some pages included too much information, and it was therefore hard for patients to follow. The professionals noticed that some links were not working, and some felt that there were too many links. The layout of the website was considered old fashioned, and the professionals proposed that it should be updated and clarified. They also recommended that more tasks especially related to patients with psychosis should be added, which could increase patients’ illness recognition.

**Table 5:** Feedback on the website.

Dimension of the feedback		Patients (N=29), n (%)	HCPs^a^ (N=28), n (%)
**Content of the website**			
	Very good	5 (17)	3 (11)
	Good	20 (69)	21 (75)
	Not good or poor	4 (14)	4 (14)
	Poor	0 (0)	0 (0)
	Very poor	0 (0)	0 (0)
**Layout of the website**			
	Very good	2 (7)	1 (4)
	Good	18 (62)	17 (61)
	Not good or poor	6 (21)	7 (25)
	Poor	3 (10)	3 (11)
	Very poor	0 (0)	0 (0)
**Usability of the website in patient education**			
	Very good	4 (14)	2 (7)
	Good	18 (62)	19 (68)
	Not good or poor	6 (21)	3 (11)
	Poor	0 (0)	4 (14)
	Very poor	0 (0)	0 (0)

^a^HCPs: Health care professionals.

### The Preliminary Impact of the Web-Based Course on Patients’ Self-Efficacy, Self-Esteem, Illness Cognition, and Knowledge About Schizophrenia

The preliminary impact of the Web-based intervention was measured at 3 time points (baseline, 8 weeks, and 6 months). Multimedia Appendix 1 shows the results of the hierarchical linear mixed models for repeated measures. Overall, there were no significant differences in time-by-group interaction with any instrument measured in this study. In a more detailed examination, we found that patients’ self-efficacy scores increased in the intervention group (at baseline: mean 26.12, SD 5.64), after the intervention (8 weeks: mean 26.50, SD 7.20), and after 6 months (mean 29.24, SD 6.05). The self-efficacy scores also increased in the control group (at baseline: mean 27.26, SD 9.36), after 8 weeks (mean 31.69, SD 6.60) but not after 6 months (mean 30.80, SD 6.41). The change between baseline and the 6-month follow-up was statistically significant in the intervention group (*P*=.003) but not in the control group. Also, the effect size (*d*=0.53) refers to medium effect in the intervention group and small in the control group (*d*=0.44). There were no statistical differences in patients’ self-esteem in either group during the 6-month study period.

The subscale *helplessness* in illness cognition decreased in the intervention group (at baseline: mean 2.26, SD 0.96) after the intervention (mean 2.11, SD 0.72) and after 6 months (mean 1.85, SD 0.59). The change between baseline and 6 months was statistically significant (*P*=.03). Also, the effect size (*d*=0.51) refers to medium effect in helplessness in the intervention group and small in the control group (*d=*0.17). The change in the control group was not significant, and there were no significant differences in the intervention group or the control group regarding the other subscales. Further, the knowledge level of patients in the intervention group increased (at baseline: mean 11.39, SD 4.65) after the intervention (mean 12.50, SD 5.26) and after 6 months (mean 15.06, SD 5.26). The change between baseline and 6 months was statistically significant (*P*=.002). The knowledge level of the control group stayed stable. Also, the effect size (*d*=0.74) refers to medium effect in the intervention group and small in the control group (*d=*0.07).

## Discussion

### Principal Findings

The aim of our study was to test the feasibility and acceptability of a Web-based patient education intervention and to report preliminary evidence of its impact on patients with schizophrenia. The feasibility was assessed by participants’ commitment to the study. We found that, in general, during the recruitment period, patients’ refusal rates were high (69% in the intervention group and 76% in the control group), which is congruent with previous studies on patients with schizophrenia [30,37-39] even though variety exists [36]. The reasons for refusal were not asked about, as study participation was voluntary, based on ethical guidelines [50], and we did not have consent to collect that information. However, the researcher visited the study wards regularly and discussed practical issues of the study with the professionals and patients. Some patients may have been concerned about the aim of the study and were therefore suspicious of it. For example, they might have been apprehensive about the confidentiality related to the research [74-76]. Previous studies have found that suspiciousness of studies and/or researchers can be one of the reasons for a high refusal rate, as it is known to be one of the symptoms of schizophrenia when the patient is in psychosis [2,74,77,78]. Even though many patients have had positive experiences with technology use [79], some patients might be afraid to engage themselves with such a study if they have difficulties with concentration [80] or they think their inpatient stay will only last for a short period of time. Moreover, some patients also think that they cannot participate in a study because they do not have schizophrenia (also [77,81]), a diagnosis that can be difficult and time consuming for some patients to accept [2]. According to a study by Woodall et al [23], the timing of asking for consent can also affect a patient’s decision to participate or not. In our study, recruitment often took place soon after a patient had been admitted to the hospital. It is therefore possible that this was too soon for some patients, because in the beginning of care, illness may be in an acute phase. There might also be patients who are wary of trying new types of treatment and would rather concentrate on proven traditional methods without any extra distraction.

High refusal rates can also be the result of professionals’ involvement, which was found to be the case in a study by Jørgensen et al [82]. Professionals may question whether their patients are too severely ill to participate or to make the decision to participate [81,82]. Therefore, in our study, the researcher reminded the professionals about recruitment and encouraged them to invite patients to participate when visiting the study wards. It is also possible that some patients refused because they did not get enough detailed information about the study. Study recruitment and studies can sometimes be seen as an extra task not related to basic nursing care. Another reason for refusals could be that there were some inconsistencies in the study recruitment; out of 303 eligible patients, there were a total of 26 patients whose willingness to participate was not asked.

The attrition rate was high in both groups (see also Kannisto et al [30]). Participation was voluntary, and reasons for deciding to dropout were not questioned, based on ethical guidelines and principles [48,49]. However, we can suggest some explanations for the dropouts that did occur. For example, some participants were discharged from the hospital before the follow-up, which made contact with them more challenging than it was with those who were able to participate in the follow-up during their hospital stay. Even though the researcher tried to contact all of them by phone and by sending them the follow-up questionnaires at home, not all had a phone, some did not answer the phone, a phone number was not in use, and 1 had a call blocker set up. In addition, some patients did not have a permanent home address or their address was unknown. Therefore, some of the reasons for dropout can be said to be because of the service system, which makes it difficult to maintain contact with the patients. However, other reasons for dropout may relate to a patient’s mental status and include lack of interest and tiredness. Patients can express interest in having something to do in the hospital, but then not actually have the time to participate [75,77]. This notion has been mentioned in a study by Furimsky et al [81], when patients with psychosis did not want to continue the study because it took up their time or they felt that their well-being had increased during the follow-up period and therefore did not think that they would benefit from participating any more. It is also possible that, in our study, patients were not willing to think about their disorder anymore or thought that, because of improved well-being and being discharged from the hospital, they were not suitable for the study anymore.

On the other hand, out of 33 participants, 31 (94%) finished all 5 intervention sessions in the patient education. This result strengthens the results of earlier studies where patients with schizophrenia spectrum disorders have engaged in Web-based patient education [23,32]. Further, Villeneuve et al [8] showed that the dropout rates from psychosocial treatments are low (13%) among patients with schizophrenia spectrum disorders. Our finding may indicate that, as soon as the participants were engaged in the study, they accepted and wanted to join the sessions. This result is supported by the evaluation of each session. The patients and the professionals alike gave positive feedback on the patient education sessions; the mean scores of the SEQ were over the midpoint of 4.0 regarding the global item *bad-good* and the subscales *depth*, *smoothness*, and *positivity*. When the session evaluations of the patients and the professionals were compared, the patients were shown to be even more convinced than the professionals that the sessions were good and deep. On the other hand, according to the professionals’ evaluations, their moods after the sessions were more positive than the moods of the patients. In a study by Kivlighan et al [83], clients’ evaluations were similarly more positive in depth and smoothness and therapists’ evaluations were stronger in positivity. This does not seem to be a general trend, however, when the evaluations between therapists and clients have varied [58,84].

In this study, to ensure the patient orientation of the intervention, the participants decided the order of the themes of their sessions. Therefore, it is not possible to directly compare the sessions and evaluate the differences between them. However, it is possible that the patients chose the theme most important to them as the theme for their first sessions and also evaluated those sessions with the highest scores. Patients might have been especially interested in, for example, patients’ rights if they were unaware of their own illness or had been involuntarily admitted. The topic *mental disorder* could have been uninteresting or unimportant to a patient if they did not consider themselves to be suffering from the disorder. Therefore, in the future, more attention should be given to the order and evaluations of the sessions. Notably, results of the subscale *arousal* need to be interpreted with caution because of the heterogeneity of the items in the scale. The feedback on the website was also positive regarding its content, layout, and usability. The positive perception of the website may help its use in the future.

Our preliminary results did not find any statistically significant differences between the intervention and control groups, indicating that the intervention did not have an impact on participants’ self-efficacy, self-esteem, illness cognition, or knowledge level any more than care as usual. On the other hand, we found that patients’ self-efficacy and knowledge levels between baseline and 6 months in the intervention group improved, while no improvement was seen in the control group. This may indicate positive outcomes of our educational intervention, although, most probably due to insufficient statistical power, not statistically different compared with care as usual. The results might also suggest that discharging a patient from the hospital between baseline and the follow-up may be a confounding factor, which could explain the positive course of self-efficacy and mental health after discharge from psychiatric hospital [85,86]. Further, we found that the knowledge of the participants improved after the Web-based patient education intervention, which has been supported in earlier studies [23].

The subscale *helplessness* in illness cognition decreased significantly in the intervention group, but not in the control group, between baseline and 6 months. In addition, self-esteem increased in both study groups, but the difference was not significant. There is little previous knowledge of how Web-based patient education impacts patients’ illness cognition and self-esteem, and therefore, more research with bigger sample sizes is needed.

Most of the patients (70% of the intervention group and 71% of the control group) used the internet, and only a few (8% of the intervention group and 9% of the control group) had negative attitudes about the internet. These findings are similar to earlier studies concerning internet use [17] and attitudes toward it [16] among Finnish patients with schizophrenia. The patients used the internet for diverse purposes, just as the rest of internet users in the Finnish population [29]. However, on the basis of the patients’ responses, even if their attitude toward the internet was mostly positive, their internet skills varied. We may therefore ask whether patients have enough confidence to use information technology as part of their treatment. To increase patients’ confidence, we should improve their skills with training by using the internet with them to ensure their computer and internet skills. Another concern is whether professionals are really ready to apply information technology in daily treatment practices [87,88]. Still, most patients and professionals found the content, layout, and usability of the website to be good.

In the control group, there were over twice as many male participants as female participants (18 vs 6). In the intervention group, the difference was minor (18 vs 15). In Finland, 48% of patients in inpatient psychiatric care are male, even though the number of male patients is greater than that of female patients among patients of working age [89]. Therefore, we may ask if female patients were more interested in participating in the intervention group than in the control group. It is therefore possible that female patients were more interested in participating in the intervention than male patients. These are important aspects when planning future interventions and studying them on a larger scale.

### Limitations

There are some limitations to this study. First, the patient attrition rate was high; we lost many participants after their discharge. Therefore, a hierarchical linear mixed model was used for the data analysis of the preliminary impacts because it allows subjects to have missing values, and we were able to use all data including subjects with missing values. Still, more detailed and systematic information about the number of these patients and reasons for not being able to contact them would be beneficial to include in future studies to get more specific information about attrition when planning new studies with bigger samples and enhanced statistical power. We now also have a hint that this type of Web-based education as a patient intervention should be designed in collaboration with inpatient and outpatient psychiatric services. Second, the sample size was small, which means that the study did not have enough statistical power to demonstrate effects of the intervention or differences between the study groups significantly and trustworthily. Owing to the underpowered sample size, study results must be considered carefully. Possible impacts of the intervention should be measured in a future study with a sufficient sample size that could compare the results of patients in rehabilitation and acute wards. Also, the instruments used for self-efficacy and self-esteem are generic [63,65] and not specific for patients with mental disorders, which might be another explanation for why improvements were not detected. Third, the last follow-up measurement was 6 months from the baseline. It is uncertain whether the changes were only due to the intervention or if they had been influenced by the time elapsed with other possible factors affecting the results [90]. Therefore, studies with a more robust design are needed in the future. In this task, we received many important improvement strategies that can be used to develop this kind of study. Fourth, owing to the quasi-experimental study design, participants were not randomized into the groups. Selection bias was minimized with baseline measurements, where we did not find differences between the groups [46]. Fifth, patients in the control group continued their treatment with care as usual without having any psychoeducational intervention from the research. Psychoeducation is, however, part of the schizophrenia care guideline in Finland [10], and it is possible that patients in the control group received some information from the professionals about their illness. Finally, the Cronbach alpha of the SEQ was low, especially in the subscale *arousal* calculated from both groups (patients and professionals), indicating that not all the participants understood the subscale items the same way. Therefore, the results regarding this subscale should be interpreted with caution. In future studies, the possibility of only using 3 subscales could be discussed [91].

### Conclusions

Although the feasibility of the intervention was poor given high refusal and dropout rates, we found that the acceptability of the intervention was good in terms of session completion. Also, the feedback on the sessions and website was positive. Furthermore, the Web-based intervention showed promising impacts on patients’ self-efficacy and knowledge levels in the intervention group. There is, therefore, potential to empower patients in the use of Web-based patient education, in terms of increased self-efficacy and illness cognition. However, a future study of this target group will require more effective strategies for recruitment, motivating patients in participation, and monitoring to decrease dropouts, especially when patients leave the hospital during the follow-up period.

### Implications

This study revealed important information about numbers of refusal and dropout and showed that more effective monitoring is needed to ensure that all possible participants are screened for eligibility and asked to participate in studies such as this. In the recruitment process, it means, for example, closer cooperation with the contact persons in the study wards during the research, but also in advance, to ensure that the recruitment process is implemented smoothly from the very beginning. Furthermore, the schedule for the researcher’s visits to the study wards could be made available in advance to staff members, possible participants, and patients who are already recruited. They would then have easy access to the researcher for asking questions before and during the research, which could improve recruitment and decrease the number of dropouts. In future studies, to increase knowledge about the subject, it might also be good to include a voluntary question asking the reasons for any refusals. Most of the patients had positive attitudes toward the internet and computers and most of them use the internet, but their skills in doing so need improving. This issue provides potential for the professionals and puts them in an important role. Therefore, it is important that professionals have the desire and the resources to support and improve patients’ internet skills to include Web-based patient education in everyday patient treatment.

## Appendix

Multimedia Appendix 1 Self-efficacy, self-esteem, illness cognition, and knowledge level at baseline, 8 weeks, and 6 months.

## Abbreviations

GSE: General Self-Efficacy Scale
ICD-10: International Classification of Diseases, 10th Revision
SEQ: Session Evaluation Questionnaire (Form 5)
SES: Rosenberg Self-Esteem Scale
