# Supplementary material for: Feasibility, Acceptability, and Preliminary Impacts of Web-Based Patient Education on Patients With Schizophrenia Spectrum Disorder: Quasi-Experimental Cluster Study
Source: J Med Internet Res. 2019 Oct 17;21(10):e13073. doi: 10.2196/13073 (PMC6913382; doi:10.2196/13073)
Supplement: Multimedia Appendix 1 [file jmir_v21i10e13073_app1.pdf]

	Intervention group								Control group								Time x group interaction
	Baseline		8 weeks		6 months				Baseline		8 weeks		6 months				
	N	Mean (SD)	N	Mean (SD)	N	Mean (SD)	Cohen *d*	*P* value	N	Mean (SD)	N	Mean (SD)	N	Mean (SD)	Cohen *d*	*P* value	*P* value
Self-efficacy	33	26.12 (5.64)	22	26.50 (7.20)	17	29.24 (6.05)	0.53	**.003**[a]	23	27.26 (9.36)	13	31.69 (6.60)	10	30.80 (6.41)	0.44	.28	.50
Self-esteem	33	28.27 (4.38)	22	28.86 (6.69)	17	29.35 (5.05)	0.23	.30	24	27.58 (8.29)	13	31.08 (7.01)	10	30.00 (6.62)	0.32	.51	.56
Illness cognition																	
Helplessness	33	2.26 (0.96)	22	2.11 (0.72)	17	1.85 (0.59)	0.51	**.03**[a]	23	2.08 (0.74)	14	1.82 (0.70)	10	1.94 (0.92)	0.17	.85	.37
Acceptance	33	2.64 (0.68)	22	2.60 (0.68)	17	2.62 (0.67)	0.03	.86	22	2.92 (0.85)	14	3.16 (1.03)	10	3.17 (0.54)	0.35	.96	.93
Perceived benefits	33	2.50 (0.82)	22	2.33 (0.60)	17	2.38 (0.82)	0.15	.71	23	2.53 (0.86)	14	2.51 (0.64)	10	2.83 (0.70)	0.38	.80	.49
Knowledge level	33	11.39 (4.65)	22	12.50 (5.26)	17	15.06 (5.26)	0.74	**.002**[a]	24	13.21 (4.63)	14	13.36 (5.37)	10	13.60 (6.60)	0.07	.77	.23

[a] Statistically significant difference analyzed with hierarchical linear mixed models for repeated measures.
